# Evidence that staphylococcal superantigens promote within-patient bacterial persistence following post-operative surgical site infection

**DOI:** 10.1128/iai.00407-24

**Published:** 2025-01-29

**Authors:** Karine Dufresne, Stephen W. Tuffs, Nicholas R. Walton, Katherine J. Kasper, Ivor Mohorovic, Farah Hasan, Tracey Bentall, David E. Heinrichs, Johan Delport, Tina S. Mele, John K. McCormick

**Affiliations:** 1 Department of Microbiology and Immunology, University of Western Ontario 468153 , London, Ontario, Canada; 2 Department of Biochemistry and Microbiology, University of Victoria 175101 , Victoria, British Columbia, Canada; 3 Division of Critical Care Medicine, Department of Medicine, University of Western Ontario 215470 , London, Ontario, Canada; 4 Department of Pathology, University of Western Ontario 6221 , London, Ontario, Canada; 5 Division of General Surgery, Department of Surgery, London Health Sciences Centre, University Hospital 90779 , London, Ontario, Canada; Universite de Geneve, Genève, Switzerland

**Keywords:** *Staphylococcus aureus*, superantigen, surgical site infection, bacteremia

## Abstract

*Staphylococcus aureus* is a predominant cause of post-operative surgical site infections and persistent bacteremia. Here, we describe a patient who experienced three episodes of *S. aureus* infection over a period of 4 months following a total knee arthroplasty. The initial bloodstream isolate (SAB-0429) was a clonal complex 5 (CC5) and methicillin-resistant *S. aureus* (MRSA), whereas two subsequent isolates (SAB-0485 and SAB-0495) were CC5 isolates but methicillin-sensitive *S. aureus*. The two latter isolates harbored a plasmid encoding three superantigen genes that were not present in the primary MRSA isolate. SAB-0485 and SAB-0495 both expressed the plasmid-encoded staphylococcal enterotoxin R exotoxin and demonstrated increased superantigen activity compared with SAB-0429. Compared to SAB-0429, the latter isolates also demonstrated an increased bacterial burden in a mouse bacteremia model that was dependent on increased interferon-γ production. Curing of the plasmid from SAB-0485 reduced this virulence phenotype. These findings suggest that the superantigen exotoxins may provide a selective advantage in chronic post-surgical infections.

**IMPORTANCE:**

In this study, we investigated bacterial isolates from a patient who experienced three recurrent *S. aureus* infections over a 4 month period following total knee arthroplasty. Genomic and phenotypic characterization of these isolates revealed that they all belonged to clonal complex 5, yet the latter two strains contained an additional plasmid encoding superantigen exotoxins. Subsequent experimental infection experiments in mice demonstrated that the plasmid-encoded superantigens exacerbated bacteremia by promoting liver abscess formation. These experiments suggest that despite appropriate antibiotic therapy, bacterial superantigens may be able to promote persistent infection following post-surgery.

## INTRODUCTION

*Staphylococcus aureus* is a common opportunistic pathogen capable of causing a wide array of infections ranging from skin lesions to invasive endocarditis, osteomyelitis, and bacteremia. Fatality rates for *S. aureus* bacteremia (SAB) in both community and hospital settings are approximately 20%–30% (1, 2), and methicillin-resistant *S. aureus* (MRSA) strains are of major concern as treatment with antibiotics often fails to clear the bacteria (3). *S. aureus* is also an important cause of post-surgical site infections (SSIs) where infection due to MRSA has been related to a 7-fold increased risk of death, a 35-fold increased risk of hospital re-admission, and over 3 weeks of additional hospitalization (4). Orthopedic SSIs prolong hospital stays for an average of 2 weeks, approximately doubling the rates of re-hospitalization and increasing hospital costs by ~300% (5, 6), and can result in decreased quality of life due to increased physical limitations (5). For these reasons, *S. aureus* decolonization strategies of the orthopedic surgical team and of patients have been implemented and demonstrate a reduction in the total number of surgical site infections (7–9).

*S. aureus* can survive and persist during infection by manipulating the host using multiple virulence factors (10), including a unique family of toxins called superantigens (SAgs) (11). SAgs trigger large-scale activation of T cells, and this aberrant activation can lead to an overwhelming cytokine storm disease known as toxic shock syndrome (12). During experimental bloodstream infection, *S. aureus* can survive and proliferate in organs, including the liver and kidney, and in this context, staphylococcal SAgs can dramatically enhance bacterial burden in the liver through the production of pathogenic levels of interferon-γ (IFNγ) that impede macrophage activity (13). Conversely, the role of staphylococcal SAgs during mucosal colonization is less well understood, but these toxins may function as immunological checkpoints in the nares, a major site of *S. aureus* colonization (14).

In this study, we characterized three consecutive clonal complex 5 (CC5) *S. aureus* isolates acquired from the same patient after the recurrence of an orthopedic SSI. Strains were isolated at days 19, 107, and 128 from the original surgical procedure, and the two latter strains differed from the primary isolate by the presence of a SAg-encoding plasmid. In this study, we define enhanced persistence as contributing to the bacterial burden over time. Following extensive genetic and phenotypic characterization of these isolates, including experimental bacteremia experiments, our findings suggest that the SAgs contribute to persistence of *S. aureus* bacteremia associated with SSIs.

## RESULTS

### Patient history

A patient in their 70s was admitted to London Health Sciences Centre hospital and received a total right knee arthroplasty, patellaplasty, and bone grafting of the distal femur on the right side to treat osteoarthritis. The site became infected 19 days post-surgery with an *S. aureus*-positive blood culture (strain SAB-0429), and a second surgery was performed to remove infected tissue. The patient received cefazolin and vancomycin after the second surgery. The patient re-presented at the clinic 107 days post-initial surgery with an infection in the right knee, again positive for *S. aureus* (strain SAB-0485). The patient was treated with vancomycin, sulfamethoxazole, and trimethoprim (Septra). An additional sample at the right knee was collected 118 days after the initial surgery that was positive for *S. aureus* (strain SAB-0495). The patient’s antibiotic regimen was changed to cefazolin, cloxacillin, and vancomycin, and no further cultures of *S. aureus* were recorded.

### Strain analysis indicates the patient was infected with two distinct clones of *S. aureus*

**Fig 1:**
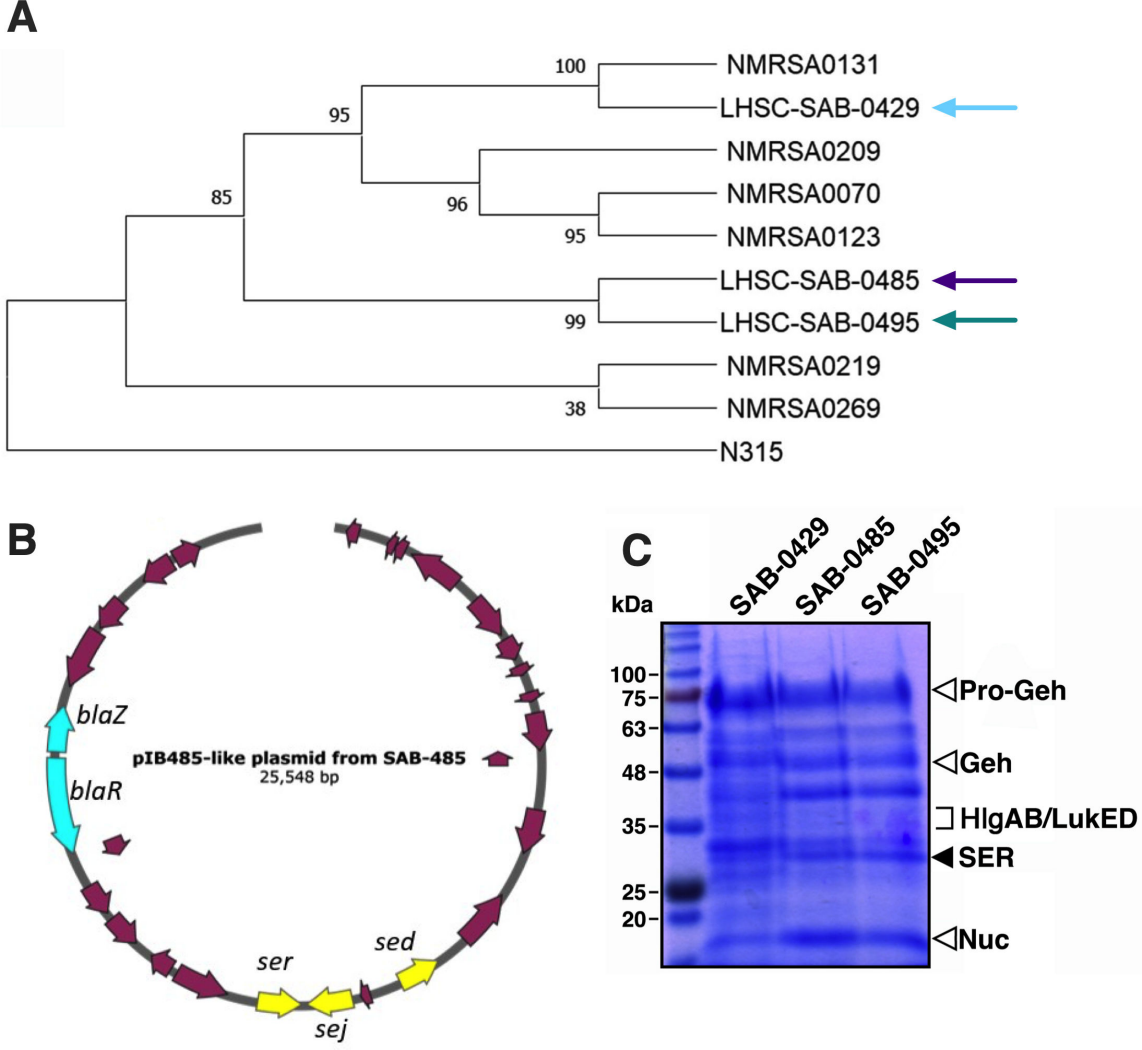
The initial methicillin-resistant *S. aureus* SAB-0429 isolate is genetically distinct from methicillin-sensitive *S. aureus* SAB-0485 and SAB-0495 isolates. (**A**) Phylogenetic tree of *S. aureus* CC5 strains including isolates SAB-0429, SAB-0485, and SAB-0495. Core single-nucleotide polymorphisms (SNPs) were identified using Snippy in the three patient isolates and a small selection of other publicly available CC5 sequences from Canada. A maximum likelihood phylogeny was constructed from the aligned SNPs using FastTree v.2.1.10. (**B**) The sequence of the pIB485-like plasmid found in both *S. aureus* SAB-0485 and SAB-0495 is presented with its main characteristics (e.g., *bla* genes and superantigen-encoding genes). (**C**) Exoprotein profiles from *S. aureus* SAB-0429, SAB-0485, and SAB-0495 visualized on SDS-PAGE. Open arrows indicated virulence factors found in all three isolates, and the solid arrow indicates SER that was only found in SAB-0485 and SAB-0495. SER, staphylococcal enterotoxin R.

We subjected the three SAB isolates to whole-genome sequencing and created *de novo* assemblies to determine genetic relationships. *In silico* multilocus sequence type analysis indicated that all three isolates were sequence type 5 (Table S1) belonging to CC5. Using a selection of publicly available CC5 sequences from Canada, we determined that, despite being of the same clonal lineage, the SAB-0429 isolate was in a distinct clade from SAB-0485 and SAB-0495 (Fig. 1A). Using the Comprehensive Antibiotic Resistance Database (CARD) (15), we found that SAB-0429 was an MRSA encoding the staphylococcal cassette chromosome *mec* element (Table S2). Curiously, the CARD analysis also identified that the SAB-0485 and SAB-0495 isolates encoded the BlaZ beta lactamase, but this was not present in the SAB-0429 isolate (Table S2). These data indicate that the patient was infected first with a clone of CC5 MRSA that was supplanted by a different CC5 methicillin-sensitive *S. aureus* (MSSA). We next compared the genome sequences of each isolate and compared them with the *S. aureus* N315 reference genome. Analysis of nucleotide differences identified 74 unique non-synonymous mutations that were present in SAB-0429 but not present in either SAB-0485 or SAB-0495. We also identified six variants that caused coding changes in both SAB-0485 and SAB-0495 but were not found in SAB-0429 (Table S3).

### SAB-0485 and SAB-0495 have a unique set of SAgs encoded on pIB485-like plasmid

To evaluate if these strains had differences in pathogenic potential, we determined the virulence factor composition present in these isolates. In reference to SAg genes, all three isolates were found to encode the staphylococcal enterotoxin genes *seg*, *sei*, *selM*, *selN*, *selW*, and *selX*; however, the *sed*, *sej*, and *ser* genes were found only in isolates SAB-0485 and SAB-0495. These three SAg genes are often found together within pIB485-like plasmids (16, 17), and this plasmid in the two MSSA isolates was confirmed from the *de novo* assembly (Fig. 1B).

To evaluate if one or all three SAgs were being expressed by SAB-0485 and SAB-0495, the extracellular proteins from the three isolates were subjected to proteomic analysis. SDS-PAGE confirmed that the secreted profiles between the MSSA and MRSA isolates were different. This was further confirmed by mass spectrometry analysis by comparison with the predicted molecule weights of the different secreted factors (Table S4). SAB-0429 produced several toxins including the pro and mature forms of glycerol-ester hydrolase, nuclease, leukocidins, and gamma hemolysins (Fig. 1C); however, no SAg peptides were detected from *S. aureus* SAB-0429. However, from SAB-0485 and SAB-0495 supernatants, the SAg staphylococcal enterotoxin R (SER) was identified (Fig. 1C). Notably, we also detected α-hemolysin (Hla) from both SAB-0485 and SAB-0495 but not SAB-0429 (Table S4).

### SAB-0485 and SAB-0495 produce increased levels of superantigen activity

**Fig 2:**
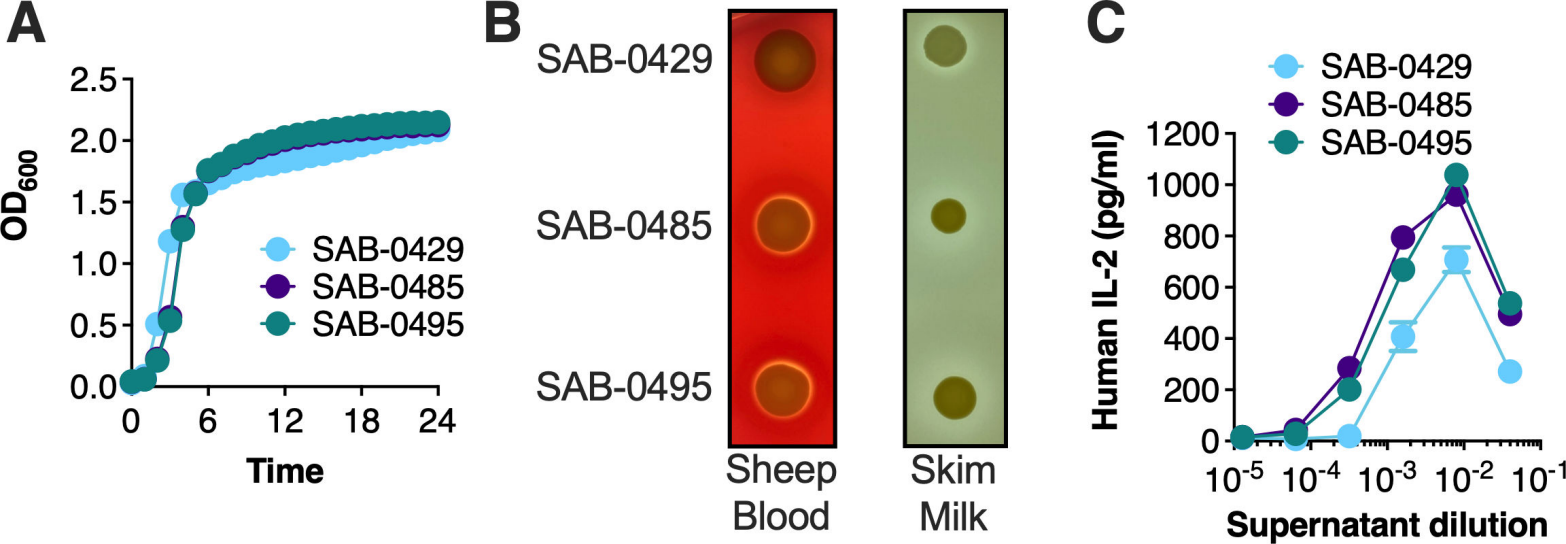
Phenotypic analyses demonstrate enhanced superantigen activity from *S. aureus* SAB-0485 and SAB-0495 compared to SAB-0429. (**A**) The three *S*. *aureus* patient isolates were grown in TSB for 24 hours with agitations in a multimode plate reader, and their optical density at 600 nm was monitored every hour. Each data point represents the mean of three independent experiments. (**B**). Hemolytic and proteolytic activities of each isolate assessed on 5% sheep blood tryptic soy agar plates and skim milk agar plate, respectively. The image is a representative image of experiments replicated at least three times. (**C**) IL-2 production from supernatants from each isolate grown in TSB for 18 hours. Supernatants were filter sterilized before exposure to human PBMCs, and IL-2 concentrations were measured by enzyme-linked immunosorbent assay. Each data point represents the mean ± SEM of three independent experiments using different donors.

To better understand how *S. aureus* may have persisted during infection, the three isolates were subjected to a range of phenotypic tests. First, growth profiles were assessed in rich bacterial media (tryptic soy broth [TSB]), and the strains grew similarly (Fig. 2A). To evaluate differences in cytolytic toxin expression, hemolytic profiles were determined by spotting the strains on tryptic soy agar containing 5% sheep blood. Hemolysis was observed for all three isolates, although this activity was decreased from the SAB-0429 isolate (Fig. 2B), consistent with an apparent reduced capacity to produce Hla (Table S3). In parallel, the isolates were spotted on skim milk plates to examine protease activity, although no differences were observed (Fig. 2B). Next, to evaluate if the additional plasmid-encoded SAgs correlated with an increased ability to induce higher levels of T-cell activation, we incubated primary human peripheral blood mononuclear cells (PBMCs) with the filter-sterilized supernatant from each isolate. Compared to SAB-0429, both SAB-0485 and SAB-0495 were able to consistently induce IL-2 at lower supernatant dilutions, indicating that the presence of the pIB485-like plasmid correlated with higher superantigenic capacity (Fig. 2C). Taken together, these analyses suggest that antibiotic treatment for the initial MRSA infection with SAB-0429 was successful, although the patient became re-infected with a persistent MSSA isolate that produced larger amounts of virulence-promoting toxins.

### Isolates containing the pIB485-like plasmid persist at a higher level in the liver

**Fig 3:**
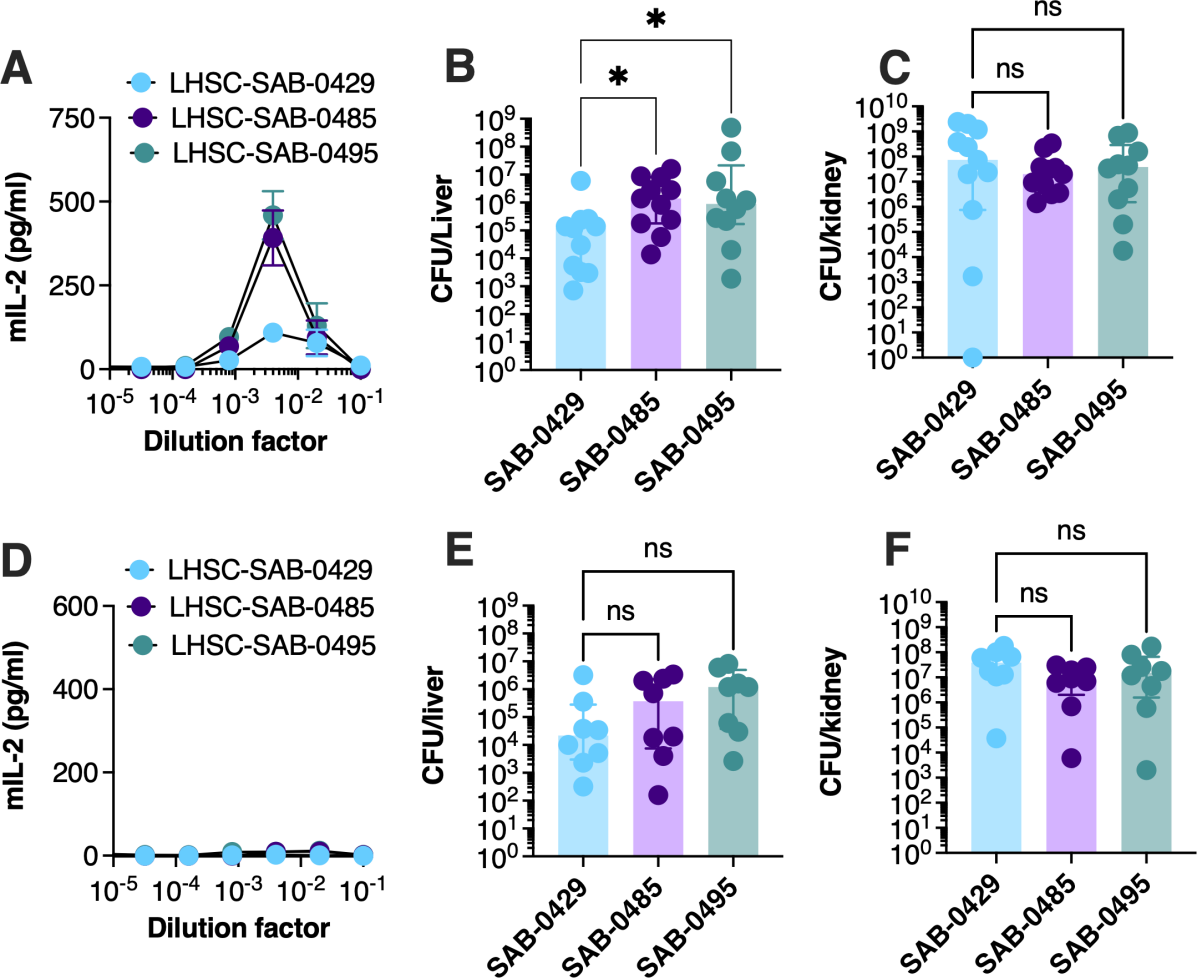
*S. aureus* isolates SAB-0485 and SAB-0495 containing the pAB485-like plasmid persist at a higher bacterial count compared to SAB-0429 in a SAg-sensitive model of bacteremia. DR4-B6 (A) or B6 (D) splenocytes were isolated and challenged against the supernatant issued from each bacterial isolate, and IL-2 production from stimulated cells was measured by enzyme-linked immunosorbent assay. Data presented are the mean of three independent experiments ± SD. Each isolate was intravenously injected at 5 × 10^7^ CFU/mL to DR4-B6 (B and C) or B6 (E and F) animals, and bacterial burden of both kidneys (B and E) and liver (C and F) was assessed 3 days post-infection. Each dot represents one animal. The results are represented as the median and interquartile range of at least eight biological replicates. Significant differences were determined using the Kruskal–Wallis test with uncorrected Dunn’s test for multiple comparisons. *P < 0.05. LHSC, London Health Sciences Centre; ns, not significant.

The increased superantigen activity of the MSSA isolates (Fig. 2C) may have promoted persistence of these strains following the second surgery. To evaluate if the SAB-0485 and SAB-0495 strains exhibited increased persistence *in vivo*, we utilized an experimental model of bacteremia in transgenic mice that express the human MHC-II molecule human leukocyte antigen (HLA)-DR4 (herein referred to as DR4-B6 mice) and are sensitive to SAg function (13, 18). The susceptibility of the DR4-B6 strain to exoproteins secreted by each *S. aureus* strain was tested by exposing extracted splenocytes with isolate supernatants and assessing for IL-2 production. Supernatants from both SAB-0485 and SAB-0495 resulted in an increased production of IL-2 compared to SAB-0429, especially observable at the dilution factor of ~1/250, noting that the more concentrated supernatants contain active cytolytic toxins that kill the immune cells (Fig. 3A). DR4-B6 mice were next inoculated intravenously with the different *S. aureus* isolates, and bacterial burden in the liver and kidneys was assessed 3 days post-inoculation. The bacterial burden in the liver of DR4-B6 mice was increased for both SAB-0485 and SAB-0495 compared with SAB-0429 (Fig. 3B), although there were no differences in bacterial counts in the kidneys (Fig. 3C). These data suggest that the SAgs encoded on the pIB485-like plasmid may be involved in promoting the persistence of the latter SAB-0485 and SAB-0495 isolates compared with the initial SAB-0429 isolate during bloodstream infection.

Hla has also been implicated in liver persistence by *S. aureus* (19). As this toxin showed increased expression from the proteomic analysis for the latter two isolates (Table S3) and that phenotypic assessment showed decreased hemolytic activity for SAB-0429 (Fig. 2B), we evaluated a potential role of Hla in the bacteremia model. Conventional mouse models are sensitive to the activity of this toxin (20); therefore, we repeated our bacteremia analysis in conventional B6 mice. Splenocytes from conventional B6 mice were first co-incubated with supernatants from each isolate which did not induce any detectable T-cell activation, confirming the lack of susceptibility of these mice to SAg (Fig. 3D). Next, the experimental bacteremia experiment was repeated in conventional B6 mice, and although there was a trend suggesting increased bacterial recovery from the liver for the two MSSA isolates, this was not statistically different (Fig. 3E). There were no differences in bacterial counts recovered from the kidneys. These collective data suggest that differences observed between the tested *S. aureus* strains were due primarily to the additional SAgs encoded by SAB-0485 and SAB-0495, rather than the expression of other virulence factors.

### Loss of pIB485-like plasmid decreases the bacterial burden in the liver

**Fig 4:**
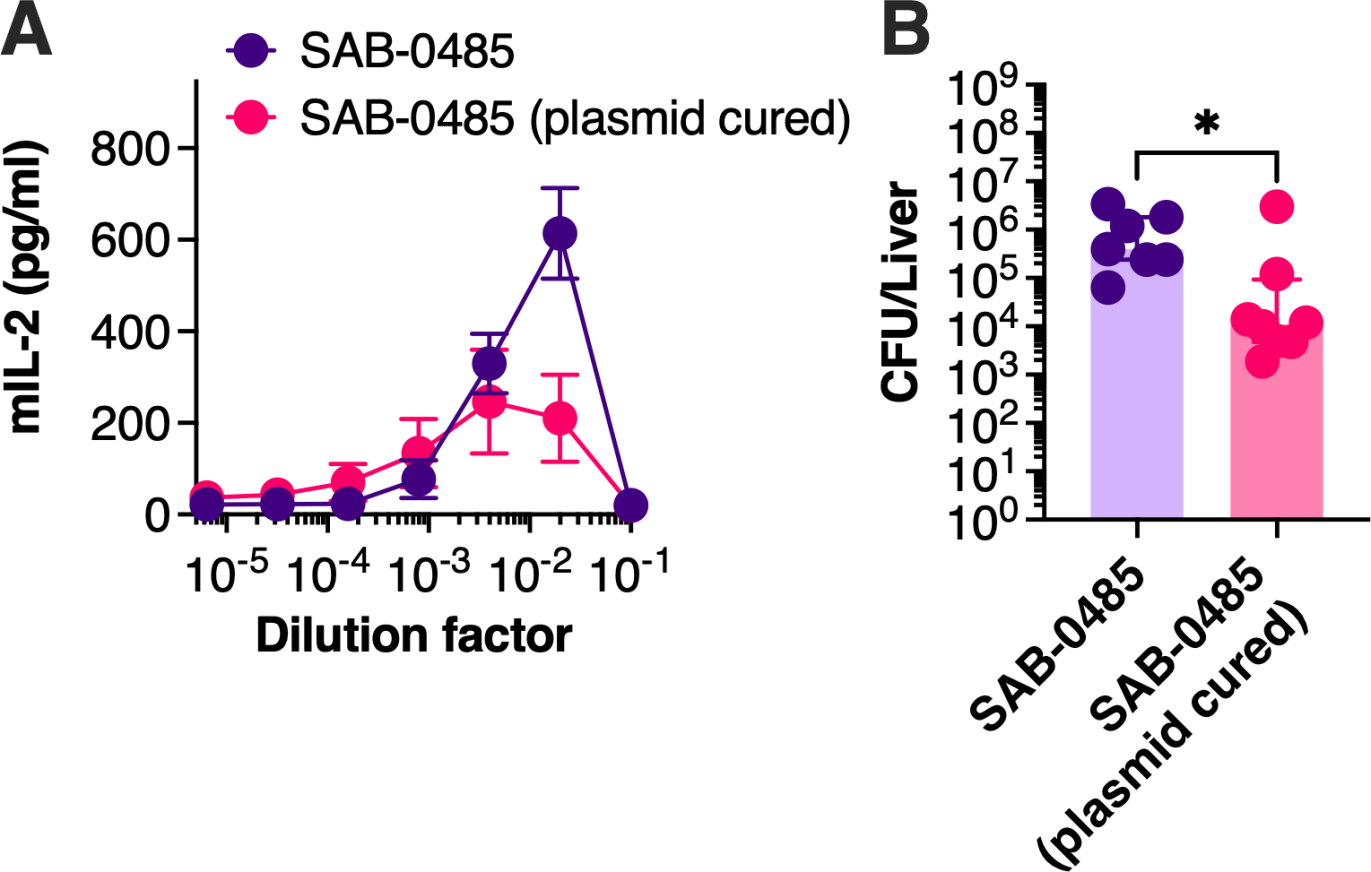
Loss of the pIB485-like plasmid in the SAB-0485 isolate demonstrates a decreased bacterial burden in the liver. (**A**) B6-DR4 splenocytes were isolated and challenged against supernatant issued from each bacterial strain (SAB-0485 or SAB-0485 without plasmid). The graph represents murine IL-2 quantification during at least three independent experiments. (**B**) The same strains were intravenously injected to DR4-B6 animals to perform the bacteremia model, and the bacterial burden in the liver is represented as the median and interquartile range of at least seven animals. Each dot represents one mouse. Significant differences were determined using Mann–Whitney test. *P < 0.05.

To determine if the increased bacterial burden within the liver of DR4-B6 mice was due to the set of SAgs encoded within pIB485-like plasmid, we cured this plasmid from SAB-0485. Multiple attempts to cure the plasmid by growing SAB-0485 at elevated temperatures were not successful, so we took advantage of the antisense *secY* counterselection system from the pKOR1 integration plasmid (21). Following integration of pKOR1 into the *ser* gene within the pIB485-like plasmid, induction of anti-*secY* successfully selected for plasmid loss, and this strain was subsequently evaluated using the bacteremia model in DR4-B6 mice. The supernatants from wild-type *S. aureus* SAB-0485 or the isogenic strain lacking the pIB485-like plasmid were tested with DR4-B6 splenocytes, and decreased IL-2 was observed with the plasmid-cured strain (Fig. 4A). We hypothesized that this decrease in T-cell stimulation would correlate with decreased bacterial burden within the liver in the DR4-B6 bloodstream infection model. Indeed, in the absence of the pIB485-like plasmid, this strain reached a lower bacterial burden in the liver of the mice compared to wild-type SAB-0429 (Fig. 4B).

### pIB485-like encoded SAgs promote a pathogenic IFNγ response

**Fig 5:**
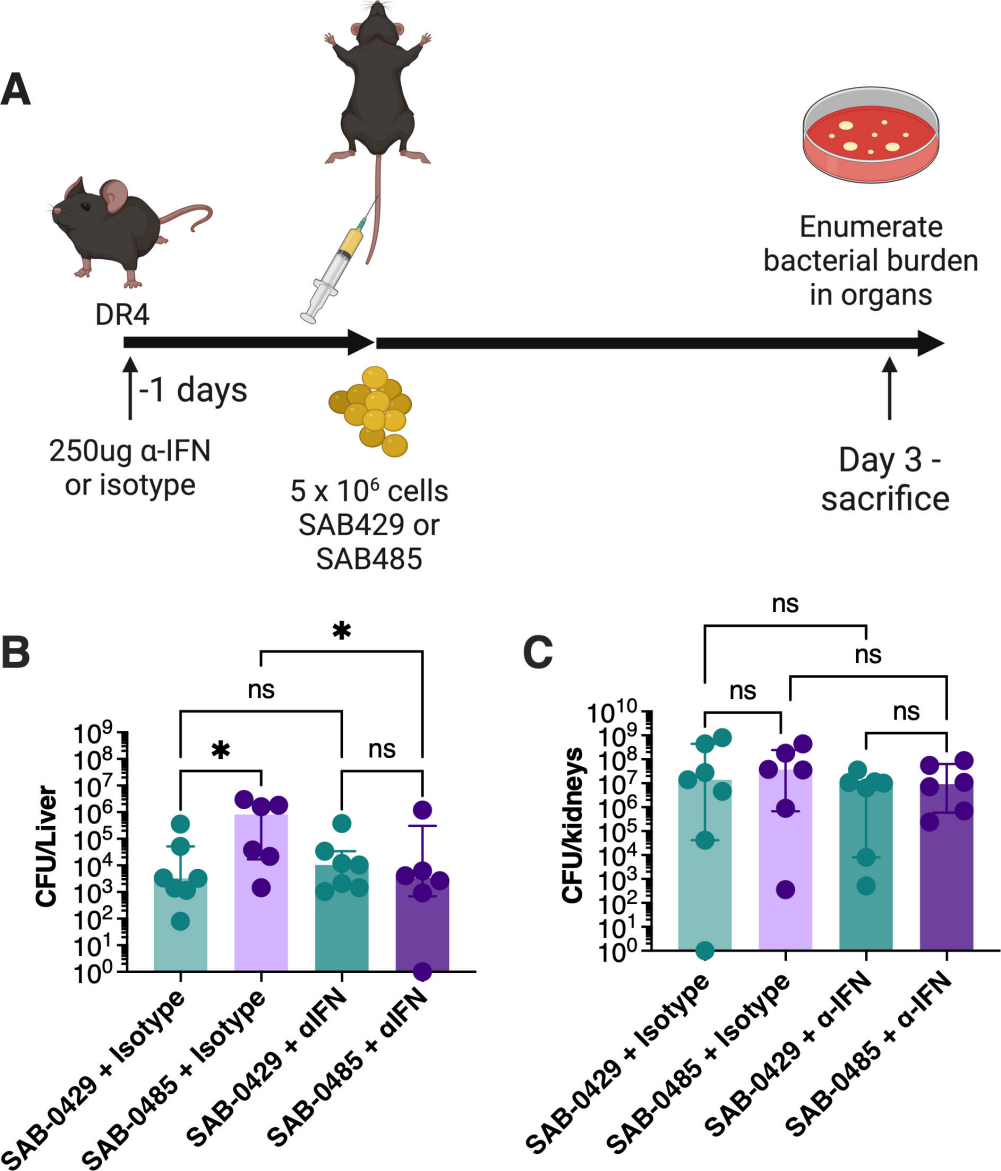
Depletion of interferon gamma eliminates SER-mediated bacterial persistence during *S. aureus* bacteremia. (**A**) The bacteremia model was repeated with the isolates SAB-0429 and SAB-0485 after intraperitoneally injecting the animal with either depleting antibodies for IFNγ or an isotype control 18 hours before *S. aureus* intravenous challenge. Both the kidneys (**B**) and the liver (**C**) were collected and homogenized for bacterial burden. The results are represented as the median and interquartile range of at least eight biological replicates. Significant differences were determined using the Kruskal–Wallis test with uncorrected Dunn’s test for multiple comparisons. *P < 0.05.

SAgs can promote a pathogenic IFNγ response that allows *S. aureus* to persist and replicate more effectively within macrophages (13). This mechanism could potentially explain why the two MSSA isolates persisted for longer in the patient compared with the MRSA clone despite antibiotic treatment. To test this hypothesis, we utilized an IFNγ depletion protocol in DR4-B6 mice (Fig. 5A). For *S. aureus* SAB-0429, depletion of IFNγ had no measurable impact on bacterial recovery from the liver or kidneys compared to the isotype antibody control. However, IFNγ depletion resulted in a significant reduction in bacterial recovery for SAB-0485 from the liver (Fig. 5B) but not the kidney (Fig. 5C). Importantly, the IFNγ depletion for SAB-0485 reduced bacterial recovery in the liver that was equivalent to the SAB-0429 isolate, indicating that removing pathogenic IFNγ production mitigated the activity of the plasmid-encoded SAgs. Together this demonstrates that the pIB485-like encoded SAgs can promote excessive IFNγ production to promote bacterial persistence during experimental bacteremia.

## DISCUSSION

*S. aureus* infections acquired in the hospital setting can result in life-threatening disease and poor outcomes, which may be further complicated by antimicrobial resistance. Additionally, microbial mechanisms that promote persistence during infection can further exacerbate disease. In this work, we encountered MSSA isolates that were able to persist in a patient despite antimicrobial therapy. Based on comparisons with the earlier MRSA isolate from the same patient and combined with experimental infection models, we found that the increased persistence of these MSSA strains was associated with the production of plasmid-encoded SAg toxins. We initially considered that this may be an example of in-patient evolution, but genome sequencing determined that although the main differentiating determinant of the strains was the pIB485-like plasmid, the SAB-0429 isolate was not ancestral to the SAB-0485 and SAB-0495 isolates (Fig. 1A). Therefore, this patient may have been colonized simultaneously at the injury site with different *S. aureus* clones or was subsequently infected with a different strain between presentations at the clinic.

All three *S*. *aureus* isolates were identified to be members of the CC5 clade. This clonal complex is clinically important and frequently isolated from nosocomial infections. In Ontario and Canada, CC5 has been related with community-associated infections, especially in the case of MRSA isolates (22). Strains from CC5 also harbor high variability in their SAg complement, including the presence or absence of the pIB485-like plasmid in both MRSA and MSSA lineages (22). Indeed, the pIB485-like plasmid contributed to this genetic variability between the isolates in this study. Interestingly, multiple experiments growing *S. aureus* SAB-0485 at elevated temperatures did not result in curing of this plasmid, suggesting this is a stable genetic element in *S. aureus* that may be associated with persistent infection and worse patient outcome.

SNP analysis revealed several differences among the three patient isolates (Table S3), but there were no differences observed in major canonical virulence regulators, including the accessory gene regulator, whose dysfunction has been often associated with MRSA isolates (23). This further suggests that the increased virulence and potential persistence of the MSSA strains are likely driven by their gene complement (specifically SAgs) rather than changes in regulatory mechanisms. Supporting this, the curing of the pIB485-like plasmid from SAB-0485 (Fig. 4) reduced this isolate virulence in our model to levels comparable to those of SAB-0429.

The MSSA isolates we identified were able to survive in the patient in between disease episodes and cause resurgence of bacteremia despite high levels of antibiotic stress. Subsequently, we were able to demonstrate that the MSSA isolates persisted in experimental *in vivo* bacteremia model, in part, by inducing a pathogenic IFNγ response (Fig. 5). We previously demonstrated that this mechanism could support the replication of *S. aureus* inside macrophages (13). Furthermore, being able to replicate within a macrophage may also effectively contribute to avoidance of killing by antibiotics. Other mechanisms of persistence within macrophages have been previously described for many bacterial pathogens; however, these mechanisms usually include a “persister-type” phenotype (24, 25). In the current study, *S. aureus* SAgs may allow survival of the bacterium independent of this persister phenotype as the quick recovery of bacteria following organ collection suggests that *S. aureus* was in a replicative state in the animal. Although only SER was detected from *in vitro* conditions by proteomics, this SAg demonstrated similar characteristics to SEA (18), SEB and SEC (13), and SE*l*-W (26) produced from other *S. aureus* strains by enhancing persistence in the liver.

Due to sampling limitations, we were unable to establish how this patient experienced infections with two distinct *S. aureus* clones. Samples at admission were not retained, so we were unable to assess if this patient was nasally colonized prior to infection, and if this was the source of the surgical complication. During the disease, co-infection with both the MSSA and MRSA clones is possible as well as hematological spread from another source. In both scenarios, a virulence mechanism that enhanced survival in macrophages would have provided a selective advantage for the MSSA strains in the patient and may have led to the clearance of the MRSA clone. Although these findings do not diminish the concerns around antimicrobial resistance, this study highlights how virulence factors may provide a selective advantage to *S. aureus* strains in certain patient settings.

## MATERIALS AND METHODS

### Bacterial strains and growth conditions

*S. aureus* strains were routinely grown aerobically at 37°C in TSB or brain heart infusion broth with appropriate antibiotics. For select experiments, isolates were spotted onto 5% sheep blood agar or 5% casein (skim milk) plates to assess hemolytic or proteolytic activity, respectively. Growth curves were performed using the Biotek Synergy H4 plate reader (Agilent). *Escherichia coli* XL1-blue was used for cloning purposes and grown aerobically at 37°C in Luria-Bertani broth with appropriate antibiotics.

### Genome sequencing

Total DNA from the *S. aureus* clinical isolates (isolates SAB-0429, SAB-0485, and SAB-0495) were sequenced using paired-end Illumina and long-read nanopore sequencing (SeqCenter, Pittsburgh, USA). Sequence data were used to generate *de novo* assemblies using SPAdes v.3.15 for chromosome and plasmid DNA and annotated using Prokka v.1.12. The assemblies have been deposited at National Center for Biotechnology Information (NCBI) (BioSamples SAMN42466856, SAMN42466857, SAMN42466858, PQ014898, and PQ014899). A core SNP alignment was built using snippy and snippy-core v.4.1 (https://github.com/tseemann/snippy) using these isolates and a selection of other publicly available CC5 sequences from Canada, and a phylogenetic tree was constructed using FastTree v.2.1.10. The presence of previously described SAgs among the genome sequence data set was established by nucleotide BLAST as implemented in blastable (https://github.com/bawee/blastable) using a threshold of 90% of identical positions to consider a gene present.

### T-cell activation assays

PBMCs were isolated as previously described (13). *S. aureus* strains were grown in TSB overnight and subcultured at 1% into fresh TSB for 18 hours, and supernatants were filter sterilized, diluted, and added to PBMCs. IL-2 concentrations were determined after 18 hours by enzyme-linked immunosorbent assay (Invitrogen).

### Proteomic analysis

Bacteria were grown in TSB overnight and subcultured in fresh TSB for 18 hours, and supernatants were harvested and normalized to an OD_600_ = 1.0. Extracellular proteins were precipitated using 6% trichloroacetic acid for 30 minutes on ice, washed in acetone, and re-suspended in 8 M urea. Resulting samples were separated on 12% acrylamide SDS-PAGE gel, and bands within a range of 15–70 kDa were identified by mass spectrometry at the London Regional Proteomics Centre. Sample spots were analyzed using a 5800 MALDI TOF/TOF System (AB Sciex) in reflectron-positive mode, and the peptide fingerprint masses were searched against the NCBI database for Gram-positive bacteria using the MASCOT search engine.

### Mice

HLA-DR4-IE (DRB1*0401) transgenic mice lacking endogenous mouse MHC-II on a C57BL/6 background (referred to as DR4-B6 mice) (19) or conventional C57BL/6 mice (referred to as B6 mice) were used for *in vivo* experiments. Mice (8–12 weeks) were sex-matched for experiments. DR4-B6 mice were bred within a barrier facility at UWO and B6 were purchased from Charles River Laboratories. Animals were housed in single-sex cages to a maximum of four animals and provided water and food *ad libitum* with appropriate environmental enrichment.

### Bacteremia infection model

Single bacterial colonies were grown overnight and subcultured at 1% into fresh TSB and grown for ~3 to 4 hours. Bacterial pellets were washed once and re-suspended in Hank’s balanced salt solution (Gibco) to an OD_600_ of 0.15 corresponding to ~5 × 10^7^ CFU/mL. Mice were injected via the tail vein with ~5 × 10^6^ CFU of *S. aureus* in a 100 µL volume. At 3 days post-infection, mice were euthanized, and kidneys and livers were harvested, homogenized, and plated on mannitol salt agar. *S. aureus* colonies were enumerated the following day with a limit of detection determined to be 3 CFU per 10 µL. IFNγ depletion experiments were performed as previously described (13).

### Curing of the pIB485-like plasmid from *S. aureus* SAB-0485

To evaluate the contribution of the pIB485-like plasmid to persistence, we attempted to cure the plasmid by repeatedly growing the strains at elevated temperatures, but these experiments did not result in plasmid loss. We therefore took a genetic approach to remove the plasmid where flanking regions of *ser* gene were PCR amplified, ligated, and cloned in pKOR1 integration plasmid within the *attP* sites of the vector as described (21). The pKOR1::*ser* plasmid was electroporated into competent SAB-0485 as described (27). Following integration of pKOR1::*ser* into the pIB485-like plasmid, bacteria were treated for 3 days with anhydrotetracycline (1 µg/mL) to induce pKOR1-encoded antisense *secY* counter selection (21), with subculturing of bacteria in fresh medium with the supplement every day. The colonies were screened for the absence of chloramphenicol resistance from pKOR1 and for the absence of ampicillin resistance from the pIB485-like plasmid. The plasmid-cured antibiotic sensitive strain was confirmed by PCR.

### Statistical analyses

Statistical analyses were performed using GraphPad Prism v.10. A *P* value equal to or lower than 0.05 was considered to be statistically significant. The bacterial burden calculated in the animal experiments was analyzed using the Kruskal–Wallis test with an uncorrected Dunn’s test for multiple comparisons or Mann–Whitney test.

## SUPPLEMENTAL MATERIAL

Supplemental material iai.00407-24-s0001.pdf Tables S1 to S4.

ASM does not own the copyrights to Supplemental Material that may be linked to, or accessed through, an article. The authors have granted ASM a non-exclusive, world-wide license to publish the Supplemental Material files. Please contact the corresponding author directly for reuse.

## References

[1] van Hal SJ, Jensen SO, Vaska VL, Espedido BA, Paterson DL, Gosbell IB. 2012. Predictors of mortality in Staphylococcus aureus bacteremia. Clin Microbiol Rev 25:362–386. doi:10.1128/CMR.05022-11 22491776 PMC3346297

[2] Lam JC, Stokes W. 2023. The golden grapes of wrath - Staphylococcus aureus bacteremia: a clinical review. Am J Med 136:19–26. doi:10.1016/j.amjmed.2022.09.017 36179908

[3] Vestergaard M, Frees D, Ingmer H. 2019. Antibiotic resistance and the MRSA problem. Microbiol Spectr 7:7. doi:10.1128/microbiolspec.gpp3-0057-2018 PMC11590431 30900543

[4] Anderson DJ, Kaye KS, Chen LF, Schmader KE, Choi Y, Sloane R, Sexton DJ. 2009. Clinical and financial outcomes due to methicillin resistant Staphylococcus aureus surgical site infection: a multi-center matched outcomes study. PLoS ONE 4:e8305. doi:10.1371/journal.pone.0008305 20016850 PMC2788700

[5] Whitehouse JD, Friedman ND, Kirkland KB, Richardson WJ, Sexton DJ. 2002. The impact of surgical-site infections following orthopedic surgery at a community hospital and a university hospital: adverse quality of life, excess length of stay, and extra cost. Infect Control Hosp Epidemiol 23:183–189. doi:10.1086/502033 12002232

[6] Hidron AI, Edwards JR, Patel J, Horan TC, Sievert DM, Pollock DA, Fridkin SK, National Healthcare Safety Network Team, Participating National Healthcare Safety Network Facilities. 2008. NHSN annual update: antimicrobial-resistant pathogens associated with healthcare-associated infections: annual summary of data reported to the National Healthcare Safety Network at the Centers for Disease Control and Prevention, 2006-2007. Infect Control Hosp Epidemiol 29:996–1011. doi:10.1086/591861 18947320

[7] Portigliatti Barbos M, Mognetti B, Pecoraro S, Picco W, Veglio V. 2010. Decolonization of orthopedic surgical team S. aureus carriers: impact on surgical-site infections. J Orthopaed Traumatol 11:47–49. doi:10.1007/s10195-010-0081-3 PMC2837811 20119678

[8] Wilcox MH, Hall J, Pike H, Templeton PA, Fawley WN, Parnell P, Verity P. 2003. Use of perioperative mupirocin to prevent methicillin-resistant Staphylococcus aureus (MRSA) orthopaedic surgical site infections. J Hosp Infect 54:196–201. doi:10.1016/s0195-6701(03)00147-6 12855234

[9] Bode LGM, Kluytmans JAJW, Wertheim HFL, Bogaers D, Vandenbroucke-Grauls CMJE, Roosendaal R, Troelstra A, Box ATA, Voss A, van der Tweel I, van Belkum A, Verbrugh HA, Vos MC. 2010. Preventing surgical-site infections in nasal carriers of Staphylococcus aureus. N Engl J Med 362:9–17. doi:10.1056/NEJMoa0808939 20054045

[10] Horváth A, Dobay O, Sahin-Tóth J, Juhász E, Pongrácz J, Iván M, Fazakas E, Kristóf K. 2020. Characterisation of antibiotic resistance, virulence, clonality and mortality in MRSA and MSSA bloodstream infections at a tertiary-level hospital in Hungary: a 6-year retrospective study. Ann Clin Microbiol Antimicrob 19:17. doi:10.1186/s12941-020-00357-z 32381015 PMC7206755

[11] Tuffs SW, Dufresne K, Rishi A, Walton NR, McCormick JK. 2024. Novel insights into the immune response to bacterial T cell superantigens. Nat Rev Immunol 24:417–434. doi:10.1038/s41577-023-00979-2 38225276

[12] McCormick JK, Yarwood JM, Schlievert PM. 2001. Toxic shock syndrome and bacterial superantigens: an update. Annu Rev Microbiol 55:77–104. doi:10.1146/annurev.micro.55.1.77 11544350

[13] Tuffs SW, Goncheva MI, Xu SX, Craig HC, Kasper KJ, Choi J, Flannagan RS, Kerfoot SM, Heinrichs DE, McCormick JK. 2022. Superantigens promote Staphylococcus aureus bloodstream infection by eliciting pathogenic interferon-gamma production. Proc Natl Acad Sci U S A 119:e2115987119. doi:10.1073/pnas.2115987119 35165181 PMC8872782

[14] Xu SX, Kasper KJ, Zeppa JJ, McCormick JK. 2015. Superantigens modulate bacterial density during Staphylococcus aureus nasal colonization. Toxins (Basel) 7:1821–1836. doi:10.3390/toxins7051821 26008236 PMC4448176

[15] Alcock BP, Huynh W, Chalil R, Smith KW, Raphenya AR, Wlodarski MA, Edalatmand A, Petkau A, Syed SA, Tsang KK, et al.. 2023. CARD 2023: expanded curation, support for machine learning, and resistome prediction at the Comprehensive Antibiotic Resistance Database. Nucleic Acids Res 51:D690–D699. doi:10.1093/nar/gkac920 36263822 PMC9825576

[16] Bayles KW, Iandolo JJ. 1989. Genetic and molecular analyses of the gene encoding staphylococcal enterotoxin D. J Bacteriol 171:4799–4806. doi:10.1128/jb.171.9.4799-4806.1989 2549000 PMC210282

[17] Omoe K, Hu D-L, Takahashi-Omoe H, Nakane A, Shinagawa K. 2003. Identification and characterization of a new staphylococcal enterotoxin-related putative toxin encoded by two kinds of plasmids. Infect Immun 71:6088–6094. doi:10.1128/IAI.71.10.6088-6094.2003 14500536 PMC201035

[18] Xu SX, Gilmore KJ, Szabo PA, Zeppa JJ, Baroja ML, Haeryfar SMM, McCormick JK. 2014. Superantigens subvert the neutrophil response to promote abscess formation and enhance Staphylococcus aureus survival in vivo. Infect Immun 82:3588–3598. doi:10.1128/IAI.02110-14 24914221 PMC4187807

[19] Surewaard BGJ, Thanabalasuriar A, Zeng Z, Tkaczyk C, Cohen TS, Bardoel BW, Jorch SK, Deppermann C, Bubeck Wardenburg J, Davis RP, Jenne CN, Stover KC, Sellman BR, Kubes P. 2018. α-Toxin induces platelet aggregation and liver injury during Staphylococcus aureus sepsis. Cell Host & Microbe 24:271–284. doi:10.1016/j.chom.2018.06.017 30033122 PMC6295203

[20] Bubeck Wardenburg J, Bae T, Otto M, Deleo FR, Schneewind O. 2007. Poring over pores: alpha-hemolysin and Panton-Valentine leukocidin in Staphylococcus aureus pneumonia. Nat Med 13:1405–1406. doi:10.1038/nm1207-1405 18064027

[21] Bae T, Schneewind O. 2006. Allelic replacement in Staphylococcus aureus with inducible counter-selection. Plasmid 55:58–63. doi:10.1016/j.plasmid.2005.05.005 16051359

[22] Guthrie JL, Teatero S, Hirai S, Fortuna A, Rosen D, Mallo GV, Campbell J, Pelude L, Golding G, Simor AE, Patel SN, McGeer A, Fittipaldi N, Investigators OCH, Delport J, Evans G, Hota S, Katz K, Lemieux C, Mertz D, Science M, Thampi N. 2020. Genomic epidemiology of invasive methicillin-resistant Staphylococcus aureus infections among hospitalized individuals in Ontario, Canada. J Infect Dis 222:2071–2081. doi:10.1093/infdis/jiaa147 32432674

[23] Butterfield JM, Tsuji BT, Brown J, Ashley ED, Hardy D, Brown K, Forrest A, Lodise TP. 2011. Predictors of agr dysfunction in methicillin-resistant Staphylococcus aureus (MRSA) isolates among patients with MRSA bloodstream infections. Antimicrob Agents Chemother 55:5433–5437. doi:10.1128/AAC.00407-11 21930887 PMC3232784

[24] Dadole I, Blaha D, Personnic N. 2024. The macrophage-bacterium mismatch in persister formation. Trends Microbiol 32:944–956. doi:10.1016/j.tim.2024.02.009 38443279

[25] Fauerharmel-Nunes T, Flannagan RS, Goncheva MI, Bayer AS, Fowler VG, Chan LC, Yeaman MR, Xiong YQ, Heinrichs DE. 2023. MRSA isolates from patients with persistent bacteremia generate nonstable small colony variants in vitro within macrophages and endothelial cells during prolonged vancomycin exposure. Infect Immun 91:e0042322. doi:10.1128/iai.00423-22 36602380 PMC9872686

[26] Vrieling M, Tuffs SW, Yebra G, van Smoorenburg MY, Alves J, Pickering AC, Park JY, Park N, Heinrichs DE, Benedictus L, Connelley T, Seo KS, McCormick JK, Fitzgerald JR. 2020. Population analysis of Staphylococcus aureus reveals a cryptic, highly prevalent superantigen SElW that contributes to the pathogenesis of bacteremia. MBio 11:e02082-20. doi:10.1128/mBio.02082-20 33109757 PMC7593966

[27] Monk IR, Shah IM, Xu M, Tan M-W, Foster TJ. 2012. Transforming the untransformable: application of direct transformation to manipulate genetically Staphylococcus aureus and Staphylococcus epidermidis. MBio 3:e00277-11. doi:10.1128/mBio.00277-11 22434850 PMC3312211

