# Supplementary material for: Evidence that staphylococcal superantigens promote within-patient bacterial persistence following post-operative surgical site infection
Source: Infect Immun. 2025 Jan 29;93(3):e00407-24. doi: 10.1128/iai.00407-24 (PMC11895439; doi:10.1128/iai.00407-24)
Supplement: Supplemental material — Tables S1 to S4. [file iai.00407-24-s0001.pdf]

SUPPLEMENTARY MATERIALS

Evidence that staphylococcal superantigens promote in-patient bacterial persistence following postoperative surgical site infection

Karine Dufresne[1*], Stephen W. Tuffs[1,2*], Nicholas R. Walton[1], Katherine J. Kasper[1], Ivor Mohorovic[1], Farah Hasan[1], Tracey Bentall[3], David E. Heinrichs[1], Johan Delport[4], Tina S. Mele[3,5], John K. McCormick[1]

Table S1. MLST and Spa typing for the *S. aureus* strains isolated from the same bacteremia patient.

	Allelic Profile							MLST	Spa
	arcC	*aroE*	*glpF*	*gmK*	*pta*	*tpi*	*yquiL*		
SAB-0429	1	4	1	4	12	1	10	ST5	t002
SAB-0485	1	4	1	4	12	1	10	ST5	t002
SAB-0495	1	4	1	4	12	1	10	ST5	t002

Table S2. Antibiotic resistance genes from genomic analyses for the *S. aureus* strains isolated from the same bacteremia patient.

		% identity		
**Gene**	**AMR Gene Family**	**SAB-0429**	**SAB-0485**	**SAB-0495**
mecR1	Methicillin resistant PBP2	100	0	0
mecI	Methicillin resistant PBP2	100	0	0
mecA	Methicillin resistant PBP2	99.7	0	0
blaZ	β-lactamase	0	93.95	93.95

Table S3. Nucleotide variations between isolates *S. aureus* SAB-0429, SAB-0485 and SAB-0495. Locus tags and nucleotide position refer to the reference genome *S. aureus* N315. Variants listed for SAB-0495 indicate variants found in SAB-0429 but not in SAB-0485 or SAB-0495. Additional variants listed for SAB-0485 or SAB-0495 were not found in SAB-0429.

LOCUS_TAG	GENE	PRODUCT	POSITION	VARIANT DESCRIPTION
***S. aureus* SAB-0429**				
SA_RS04535	*dltB*	PG:teichoic acid D-alanyltransferase DltB	900486	conservative_inframe_insertion c.586_588dupGAA p.Glu196dup
SA_RS03025	*sdrC*	MSCRAMM family adhesin SdrC	607569	disruptive_inframe_insertion c.2360_2361insTTCAGA p.Asp787_Ser788insSerAsp
SA_RS12155		alpha-glucoside-specific PTS transporter subunit IIBC	2377036	frameshift_variant c.1299dupA p.Gly434fs
SA_RS01105	*murQ*	N-acetylmuramic acid 6-phosphate etherase	218647	frameshift_variant c.157delA p.Thr53fs
SA_RS03830		biotin-dependent carboxyltransferase family protein	765019	frameshift_variant c.259_269delATATCTCCATA p.Ile87fs
SA_RS00500		K(+)-transporting ATPase subunit C	80871	frameshift_variant c.25dupG p.Asp9fs
SA_RS00500		K(+)-transporting ATPase subunit C	81246	frameshift_variant c.402delT p.Phe134fs
SA_RS00500		K(+)-transporting ATPase subunit C	81252	frameshift_variant c.408delA p.Lys136fs
SA_RS09190		DUF4888 domain-containing protein	1866625	frameshift_variant c.552dupA p.Arg185fs
SA_RS07495		DUF1672 domain-containing protein	1529843	frameshift_variant c.752delA p.Asn251fs
SA_RS12585		Txe/YoeB family addiction module toxin	2466191	frameshift_variant c.76dupA p.Met26fs
SA_RS14050		peptide-methionine (S)-S-oxide reductase	2765943	missense_variant c.109T>G p.Ser37Ala
SA_RS06060	*fakA*	fatty acid kinase catalytic subunit FakA	1209288	missense_variant c.1114A>G p.Lys372Glu
SA_RS02205		superantigen-like protein SSL4	444605	missense_variant c.118G>A p.Ala40Thr
SA_RS01315		3-hydroxyacyl-CoA dehydrogenase/enoyl-CoA hydratase family protein	269499	missense_variant c.1216A>G p.Ile406Val
SA_RS02380		sodium-dependent transporter	479435	missense_variant c.1283A>G p.Lys428Arg
SA_RS11465	*lacG*	6-phospho-beta-galactosidase	2262777	missense_variant c.1293G>T p.Trp431Cys
SA_RS07480		DUF1672 domain-containing protein	1526328	missense_variant c.134G>A p.Gly45Asp
SA_RS07105		S41 family peptidase	1427728	missense_variant c.1360C>A p.Gln454Lys
SA_RS08880		polysaccharide biosynthesis protein	1805530	missense_variant c.1639C>T p.Arg547Cys
SA_RS06270		DNA polymerase III subunit alpha	1255712	missense_variant c.1835T>G p.Val612Gly
SA_RS05535	*isdB*	heme uptake protein IsdB	1104746	missense_variant c.1850C>T p.Ser617Leu
SA_RS02205		superantigen-like protein SSL4	444673	missense_variant c.186_187delGCinsAT p.Pro63Ser

SA_RS11425	*sfaD*	D-ornithine--citrate ligase SfaD	2253646	missense_variant c.188C>T p.Ala63Val
SA_RS08670		GAF domain-containing protein	1760402	missense_variant c.191G>A p.Gly64Glu
SA_RS00440		DUF927 domain-containing protein	68224	missense_variant c.200A>T p.Glu67Val
SA_RS15270		hypothetical protein	1872187	missense_variant c.221A>T p.Asn74Ile
SA_RS01745		NupC/NupG family nucleoside CNT transporter	357150	missense_variant c.236G>T p.Gly79Val
SA_RS13130	*fnbB*	fibronectin-binding protein FnbB	2568615	missense_variant c.2593_2594delGTinsAC p.Val865Thr
SA_RS14020	*sasA*	serine-rich repeat glycoprotein adhesin SasA	2759358	missense_variant c.2711C>T p.Ser904Leu
SA_RS00565	*cstA*	persulfide response sulfurtransferase CstA	91794	missense_variant c.274G>T p.Gly92Cys
SA_RS03135		APC family permease	634296	missense_variant c.280G>A p.Val94Met
SA_RS15265	*ebh*	hyperosmolarity resistance protein Ebh	1440174	missense_variant c.29629A>C p.Lys9877Gln
SA_RS10545	*mroQ*	CPBP family intramembrane metalloprotease MroQ	2074787	missense_variant c.296T>C p.Val99Ala
SA_RS15265	*ebh*	hyperosmolarity resistance protein Ebh	1440008	missense_variant c.29795A>G p.Asp9932Gly
SA_RS10385		hypothetical protein	2046930	missense_variant c.29A>G p.Lys10Arg
SA_RS01615	*essC*	type VII secretion protein EssC	335639	missense_variant c.3121C>T p.His1041Tyr
SA_RS06955		ATP-binding cassette domain-containing protein	1397395	missense_variant c.316C>T p.Pro106Ser
SA_RS05135		glucosaminidase domain-containing protein	1026766	missense_variant c.3323T>C p.Ile1108Thr
SA_RS13955		PTS fructose transporter subunit IIABC	2733732	missense_variant c.346G>A p.Gly116Ser
SA_RS06415		RicAFT regulatory complex protein RicA family protein	1290191	missense_variant c.350C>T p.Ala117Val
SA_RS11455		NADP-dependent oxidoreductase	2260878	missense_variant c.367A>G p.Ser123Gly
SA_RS13200		ATP-binding cassette domain-containing protein	2587394	missense_variant c.376G>A p.Gly126Ser
SA_RS02205		superantigen-like protein SSL4	444873	missense_variant c.386C>A p.Thr129Lys
SA_RS00440		DUF927 domain-containing protein	68029	missense_variant c.395G>A p.Ser132Asn
SA_RS00440		DUF927 domain-containing protein	68010	missense_variant c.413_414delAAinsCG p.Lys138Thr
SA_RS02925		class I SAM-dependent methyltransferase	579232	missense_variant c.436_437delCGinsTC p.Arg146Ser
SA_RS14765		IS1182 family transposase	1839989	missense_variant c.448T>C p.Trp150Arg
SA_RS03755		aldo/keto reductase	753704	missense_variant c.451G>A p.Ala151Thr
SA_RS00440		DUF927 domain-containing protein	67967	missense_variant c.453_457delTAATGinsGAATA p.AspAsnAla151GluAsnThr
SA_RS00440		DUF927 domain-containing protein	67953	missense_variant c.469_471delACTinsGCA p.Thr157Ala
SA_RS07710		membrane protein	1570893	missense_variant c.46G>A p.Gly16Arg
SA_RS00440		DUF927 domain-containing protein	67944	missense_variant c.476_480delAAGGTinsGGGGA p.Lys159Arg
SA_RS14130	*hisB*	imidazoleglycerol-phosphate dehydratase HisB	2780129	missense_variant c.488C>A p.Ala163Asp
SA_RS08420	*hemA*	glutamyl-tRNA reductase	1704687	missense_variant c.494T>C p.Val165Ala

SA_RS06185	*dprA*	DNA-processing protein DprA	1236148	missense_variant c.4A>G p.Ile2Val
SA_RS09635		phosphoglycerate dehydrogenase	1926062	missense_variant c.56G>A p.Arg19His
SA_RS13395	*feoB*	ferrous iron transport protein B	2624787	missense_variant c.580T>C p.Tyr194His
SA_RS03280		iron ABC transporter permease	661713	missense_variant c.604T>A p.Leu202Ile
SA_RS04940		MFS transporter	990293	missense_variant c.613G>A p.Asp205Asn
SA_RS13445		D-lactate dehydrogenase	2636543	missense_variant c.61G>C p.Ala21Pro
SA_RS04905	*mgtE*	magnesium transporter	981189	missense_variant c.673G>T p.Asp225Tyr
SA_RS13035		hypothetical protein	2546892	missense_variant c.73G>A p.Glu25Lys
SA_RS05895	*ileS*	isoleucine--tRNA ligase	1172112	missense_variant c.803C>T p.Ser268Phe
SA_RS13805		CitMHS family transporter	2703993	missense_variant c.816A>G p.Ile272Met
SA_RS06845	*trpB*	tryptophan synthase subunit beta	1377769	missense_variant c.823C>T p.Leu275Phe
SA_RS10310		AAA family ATPase	2040054	missense_variant c.829C>T p.Arg277Cys
SA_RS13955		PTS fructose transporter subunit IIABC	2734245	missense_variant c.859G>T p.Ala287Ser
SA_RS13690	*panB*	3-methyl-2-oxobutanoate hydroxymethyltransferase	2680602	missense_variant c.97G>T p.Ala33Ser
SA_RS08750	*harA*	haptoglobin-binding heme uptake protein HarA	1776769	stop_gained c.1187G>A p.Trp396*
SA_RS10555		nitroreductase family protein	2076915	stop_gained c.212G>A p.Trp71*
SA_RS01775		YjiH family protein	365000	stop_gained c.41G>A p.Trp14*
SA_RS06560	*cls*	cardiolipin synthase	1316820	stop_gained c.792G>A p.Trp264*
SA_RS13250		NAD(P)H-dependent oxidoreductase	2596639	stop_gained c.85A>T p.Lys29*

***S. aureus* SAB-0485 and SAB-0495**

SA_RS03030	*sdrD*	MSCRAMM family adhesin SdrD	612222	disruptive_inframe_deletion c.3789_3794delTTCAGA p.Ser1264_Asp1265del
SA_RS10550		SdrH family protein	2076124	disruptive_inframe_insertion c.383_384insGGATCCAAAACC p.Pro128_Asp129insAspProLysPro
SA_RS10180		HK97 gp10 family phage protein	2025528	missense_variant c.127G>A p.Val43Ile
SA_RS01670		TIGR01741 family protein	344035	missense_variant c.340C>T p.Leu114Phe
SA_RS10155		phage tail tape measure protein	2022893	missense_variant c.398G>A p.Arg133Lys
SA_RS00930		glycosyltransferase	175721	missense_variant c.431T>A p.Val144Glu

Table S4. Proteomic analysis of the supernatants from the *S. aureus* strains isolated from the same bacteremia patient.

Strain	Spot #	Predicted Size (Da)	Protein identified	Protein Score
SAB-0429	0429-15	23949	Thermonuclease [*S. aureus* subsp. *aureus* CIG1750]	142
	0429-20	18358	Alkyl hydroperoxide reductase, partial [*S. aureus*]	117
	0429-25	22375	Superantigen-like protein (Sav0433) from *S. aureus* Mu50	132
	0429-28	32220	Glycerophosphodiester phosphodiesterase, partial [*S. aureus*]	147
		34968	Gamma-hemolysin subunit A [*S. aureus*]	139
	0429-35	34028	Chain A, leukocidin F (Hlgb) from *S. aureus*	113
		32090	Pyridoxal biosynthesis protein [*S. aureus*]	95
		31410	Leukotoxin LukD [*S. aureus* DAR5844]	150
		27094	Gamma-hemolysin protein B [*S. aureus*]	136
	0429-70	71387	Lipase [*S. aureus*]	178
SAB-0485	0485-15	25216	Thermonuclease [*S. aureus*]	144
	0485-20	10311	50S ribosomal protein L6, partial [*S. aureus* M0271]	101
	0485-25	30023	Enterotoxin [*S. aureus*] (SER)	119
	0485-28	32220	Glycerophosphodiester phosphodiesterase [*S. aureus*]	132
		33115	Chain B of octameric pore of gamma-hemolysin from *S. aureus*	113
		36389	Leukotoxin LukE [*S. aureus* LPIH6021]	121
		36349	Gamma-hemolysin component A [*S. aureus* VRS2]	109
	0485-30	34437	Chain A, crystal structure of alpha-hemolysin	174
	0485-35	34028	Chain A, leukocidin F (Hlgb) from *S. aureus*	159
		36429	Gamma-hemolysin component B [*S. aureus* CIG1835]	155
		31410	Leukotoxin LukD [*S. aureus* DAR5844]	147
		35244	Chain A, of octameric pore of gamma-hemolysin from *S. aureus*	125
	0485-70	76495	Lipase [*S. aureus*]	271
SAB-0495	0495-15	23949	*S. aureus* sub. sp. *aureus* thermonuclease	123
	0495-20	12679	*S. aureus* 50s ribosomal protein L6	89
	0495-25	30023	Enterotoxin (SER)	110
	0495-28	32220	Glycerophosphodiester phosphodiesterase [*S. aureus*]	169
		33115	Chain B of octameric pore form of gamma-hemolysin, *S. aureus*	127
	0495-30	34437	Chain A of Alpha-Hemolysin	146
	0495-35	34028	Chain A, leukocidin F (Hlgb) from *S. aureus*	118
		36698	Gamma-hemolysin subunit B [S. aureus]	113
		34870	Chain B, covalent S-f heterodimer of Gamma-hemolysin	102
		31410	Leukotoxin LukD [*S. aureus* DAR5844]	97
		35244	Chain A, of octameric pore of gamma-hemolysin From *S. aureus*	97
		27094	Gamma-hemolysin protein B [*S. aureus*]	89
	0495-70	76486	Lipase [*S. aureus*]	203
